# Modification of mesenchymal stem cells for cartilage-targeted therapy

**DOI:** 10.1186/s12967-022-03726-8

**Published:** 2022-11-08

**Authors:** Jianghong Huang, Qisong Liu, Jiang Xia, Xi Chen, Jianyi Xiong, Lei Yang, Yujie Liang

**Affiliations:** 1 Department of Spine Surgery and Orthopedics, Shenzhen Second People’s Hospital (First Affiliated Hospital of Shenzhen University, Health Science Center), Shenzhen, 518035 China; 2 Innovation Leading Engineering Doctor, Tsinghua University Shenzhen International Graduate School, Class 9 of 2020, Shenzhen, 518055 China; 3 Department of Spine Surgery and Institute for Orthopaedic Research, the Second Clinical Medical College of Jinan University (Shenzhen People’s Hospital), Shenzhen Key Laboratory of Musculoskeletal Tissue Reconstruction and Function Restoration, Shenzhen, 518020 China; 4 Department of Chemistry, the Chinese University of Hong Kong, Shatin, Hong Kong SAR, China; 5 Department of Orthopaedics, The Second Affiliated Hospital of Shenzhen University (People’s Hospital of Shenzhen Baoan District), Shenzhen, 518000 China; 6 Department of Orthopedics, Affiliated Hospital of Jining Medical University, Jining Medical University, Shandong, China

**Keywords:** Osteoarthritis, Mesenchymal stem cells, Targeted delivery

## Abstract

Osteoarthritis (OA) is a chronic degenerative joint disease characterized by the destruction of the articular cartilage, sclerosis of the subchondral bone, and joint dysfunction. Its pathogenesis is attributed to direct damage and mechanical destruction of joint tissues. Mesenchymal stem cells (MSCs), suggested as a potential strategy for the treatment of OA, have shown therapeutic effects on OA. However, the specific fate of MSCs after intraarticular injection, including cell attachment, proliferation, differentiation, and death, is still unclear, and there is no guarantee that stem cells can be retained in the cartilage tissue to enact repair. Direct homing of MSCs is an important determinant of the efficacy of MSC-based cartilage repair. Recent studies have revealed that the unique homing capacity of MSCs and targeted modification can improve their ability to promote tissue regeneration. Here, we comprehensively review the homing effect of stem cells in joints and highlight progress toward the targeted modification of MSCs. In the future, developments of this targeting system that accelerate tissue regeneration will benefit targeted tissue repair.

**Graphical Abstract:**

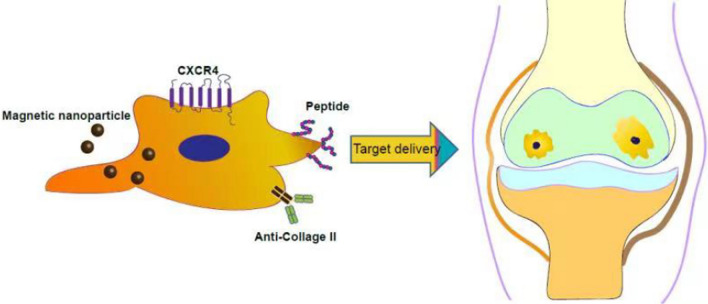

## Introduction

Osteoarthritis (OA) is a chronic joint disease characterized by articular cartilage (AC) degeneration and subchondral bone hyperplasia. OA is one of the leading causes of disability in the elderly and during adulthood. As the elderly population increases over the coming decades, the prevalence of arthritis will also increase. Currently, treatment options for OA are limited to pain relief and joint replacement surgery [1]. Targeted repair of the damaged cartilage and restoration of joint function is critical to treating osteoarthritis. Cell therapy, especially transplantation of mesenchymal stem cells/stromal cells (MSCs), represents an effective solution for tissue regeneration and repair as these pluripotent stem cells have the potential to differentiate into cartilage. MSCs can be used as seed cells that directly participate in the local repair and regulate metabolism and immune function through their secretory functions, thereby preventing or delaying the need for joint replacement surgery. Given their versatility, MSCs may play key roles in different stages of cartilage repair. Moreover, because the joint cavity is a relatively closed space and can be easily targeted by injection, the feasibility of injecting MSCs to treat joint diseases is significant compared to treating diseases that require systemic MSC injection. Several pre-clinical and clinical studies of MSC therapy in OA have indicated its safety and reliability [2–4]. However, the ability to effectively deliver exogenous MSCs to the injury site for enhancing the regeneration process remains an outstanding challenge for cell therapy. Although intra-articular (IA) injection of ex vivo expanded MSCs can increase the number of cells in the joint cavity, most MSCs fail to migrate toward the injured area. Due to the low targeting efficiency of MSCs, current MSC-based regenerative therapies require IA injection of a large number of cells.

Advances in biology, genetic engineering, and chemical biology related to MSCs have opened up new avenues for enhancing the efficacy of MSCs in treating OA. Targeted modification of MSCs can enhance their migration potential and facilitate their homing ability, translating preclinical research into effective and safe targeted therapies.

Here, we review recent advances in targeted cell therapy to repair of articular cartilage (AC) tissue in OA, with special attention to AC. We describe the structure and function of healthy and transparent AC and OA and current strategies for enhancing the delivery of MSCs to cartilage tissue. We focus primarily on targeting methods, including cell surface modifications and magnetic-assisted tissue targeting, discuss their advantages and limitations, provide additional perspectives, and examine emerging strategies based on new research findings. These findings are being verified in preclinical models, which are expected to develop into early proof-of-concept trials and provide information for designing future definitive clinical trials.

### Stem cells in AC repair

Unlike tissues with vascular blood supply, avascular cartilage tissue does not immediately trigger an inflammatory response upon injury, limiting its ability to promote repair and self-regeneration [5]. Therefore, endogenous cells may be recruited to diseased sites and act as key players in tissue regeneration. Stem cells (progenitor cells) with self-renewal capacity, identified in the surface regions of AC, have been designated cartilage-derived stem/progenitor cells [6]. Synovial fluid/synovial membrane MSCs have also been found to support self-repair in the joint cavity [7]. The potential applications of these cells remain unclear, but ongoing research seeks to better understand these cellular phenotypes and their therapeutic value for cartilage repair.

MSCs have the capacity for self-renewal and chondrogenic differentiation, making them an optimal cellular source for cartilage regeneration. MSCs can be derived from a variety of autologous tissues, including bone marrow (BMSCs), adipose tissue (ADSCs), synovial tissue (SDSCs), and peripheral blood (PB-MSCs) [8, 9]. Based on the specific cartilage pathology, MSCs can be implanted into the defect area after a surgical incision or administered by IA injection.

In 2008, Centeno et al. first reported the injection of autologous BMSCs in patients with degenerative cartilage disease [10]. Preliminary clinical data from Qiao et al. [59] showed no adverse events and significant therapeutic benefits after the highest dose of ADSC injection. Furthermore, the combined effect of the scaffold material has become more obvious. Hallam and colleagues seeded BMSCs with a platelet fibrin glue (FG) scaffold and demonstrated significant cartilage renewal by clinical MRI [11]. Similarly, Kuroda and coworkers implanted BMSCs onto collagen membranes, and reported a significant improvement in defects filled with hyaline-like cartilage tissue [12].

We have summarized representative current clinical uses of MSC transplantation for the treatment of cartilage injury and OA in Table 1. Regardless of cell source or implantation method, most studies corroborate the clinical benefits of MSCs in AC regeneration. However, the utility of MSCs remains debatable due to many unanswered questions. According to the International Association for Cartilage Repair criteria, 76% of patients who receive MSC implantation exhibited abnormal or severely repaired tissue upon second-look arthroscopic assessment [13]. Thus, reliable clinical confirmation of the safety and efficacy of this approach is required through double-blind, controlled, prospective multicenter studies with longer follow-up duration. Indeed, intra-articular injection of MSCs must be performed directly into the injury site to receive a therapeutic benefit. However, lack of targeting may lead to cell diffusion into non-target tissues, posing a potential barrier to the clinical translation of MCS-based cartilage therapies. Off-target effects appear to cause low engraftment. For example, in a rabbit model, MSCs were found to migrate to the upper knee, subchondral bone, and popliteal fossa following IA injection, but no MSCs were seeded in the cartilage defects [14]. Therefore, improved tracking of transplanted cells in the cartilage is needed to better understand the mechanisms underlying MSC migration and homing. The targeted engineering of MSCs promises to further improve clinical outcomes for local cartilage lesions.

**Table 1:** Summary of clinical trials of MSC-based therapy for the treatment of cartilage lesions

Cell sources	Number of Cells	Supplement with	Follow-up	Pathology type	Delivery type	Results	Publication
Autologous BMSCs	–	Platelet lysate	24 weeks	Knee cartilage defects	IA injection	Pain and motion improvement, Significant cartilage and meniscus regeneration detected by MRI	[15]
Autologous BMSCs	4 × 10^7^	–	12/24 weeks	Knee OA	IA injection	Significant improvement in MRI outcomes and clinical performance	[16]
Autologous BMSCs	–	Collagen gel; periosteum	12 months	Knee cartilage defects	Surgical delivery	Promote hyaline-like type of cartilage tissue regeneration, remarkable improvement in clinical symptoms	[12]
Autologous BMSCs	1 × 10^6^, 1 × 10^7^, 5 × 10^7^	–	12 months	Knee OA	IA injection	Reduces Synovial Inflammation, The clinical symptoms of the 5 × 10^7^ cell group were significantly improved	[17]
Autologous BMSCs	8–9 × 10^6^	–	12 months	Knee OA	IA injection	Walking time without pain improved	[18]
Autologous BMSCs	4 × 10^7^	2% human serum albumin	6 months	Knee OA	IA injection	Alleviating pain and patient symptoms	[19]
Autologous BMSCs	6 × 10^7^ ± 0.6 × 10^6^	–	24 months	Knee OA	IA injection	Clinical outcome and knee cartilage thickness were significantly improved	[20]
Autologous BMSCs	1 to 10 × 10^7^	HA	12 months	Knee OA	IA injection	Clinical and functional improvement of knee OA	[21]
Autologous BMSCs	–	Platelet-rich fibrin glue	6, 12 months	Cartilage Defects	Surgical implantation	Promote the repair of articular cartilage defects	[11]
Autologous BMSCs	1 × 10^7^, 1 × 10^8^	Fibrin glue	12 months	Knee OA	IA injection	Significant improvement in WOMAC and VAS scores	[22]
Autologous BMSCs	8–9 × 10^6^		5 years	Knee OA	IA injection	Pain and walking distance were improved, the worse of knee were delayed	[23]
Autologous BMSCs	5 × 10^6^	–	2, 6, 12, 30 months	Knee, ankle, or hip OA	IA injection	MRI showed improved VAS and WOMAC scores	[24]
Allogeneic BMSCs	1.5–2 × 10^6^	–	12 months	Knee OA	IA injection	Promote hyaline-like regeneration and clinical outcome	[25]
Allogeneic BMSCs	2.5 × 10^7^, 5 × 10^7^, 7.5 × 10^7^, 1.5 × 10^8^	–	1, 3, 6, 12 months	Knee OA	IA injection	Cell dose at 2.5 × 10^7^ was the most effective among the doses tested for pain relief and clinical score	[26]
Allogeneic BMSCs	4 × 10^7^	Hyaluronic acid	12 months	Knee OA	IA injection	Improvement in pain and cartilage quality	[27]
Allogeneic BMSCs	5 × 10^7^, 1.5 × 10^8^	Hyaluronic acid/hyaluronan	55/24	Knee OA	IA injection	Pain alleviation and MRI evidence of meniscus regeneration	[4]
Autologous ADSCs	1 × 10^7^, 2 × 10^7^, 5 × 10^7^		24 months	Knee OA	IA injection	Clinically safe and significant improvement in clinical symptoms	[28]
Autologous ADSCs	4.3 × 10^6^	Platelet-rich plasma or fibrin glue scaffold	28.6 months	Knee OA	Arthroscopic surgery	Better repair outcome and IKDC scores in the implantation MSC group on a fibrin glue scaffold	[29]
Autologous ADSCs	4.4 × 10^6^	Fibrin glue	27.9 months	Knee chondral defects	Arthroscopic implantation	Significant improvement in clinical and MRI outcomes	[30]
Autologous ADSCs	2 × 10^6^, 1 × 10^7^, 5 × 10^7^	–	6 months	Knee OA	IA injection	Low-dose ADSCs significantly improve pain and function	[31]
Autologous ADSCs	1.4 × 10^7^	–	3-months	Knee OA	IA injection	Pain relief in osteoarthritic knees, significantly improved clinical outcomes in WOMAC and VAS scores	[32]
Autologous ADSCs	1 × 10^7^, 5 × 10^7^, 1 × 10^8^	–	6 months	Knee OA	IA injection	Regeneration with hyaline‐like articular cartilage	[33]
Autologous ADSCs	5 × 10^7^	Ultrasound guidance	24-month	Knee OA	IA injection	Long-term safety and efficacy	[34]
Allogeneic hUCB-MSCs	–	–	18.7 months	Medial unicompartmental knee OA	Surgical delivery	More effective in cartilage regeneration	[35]
Allogeneic hUCB-MSCs	–	Hyaluronate	48-week	Cartilage defects	Surgical delivery	Cartilage repair, pain and function improvement	[36]
Allogeneic hUC-MSCs	2 × 10^7^	5% AB plasma	24/48 weeks	Knee OA	IA injection	Improved clinical scores and MRIs	[37]
Allogeneic hUC-MSCs	1 × 10^7^	Hyaluronic acid	6 months	Knee OA	IA injection	Improved clinical score	[38]
Allogeneic hUCB-MSCs	–	–	7 years	Knee OA	IA injection	Clinically safe and effective for cartilage regeneration	[39]
Allogeneic hUCB-MSCs	2.5 × 106 cells/cm2	4% HA (CARTISTEM®)	36.1 ± 6.4 months	Knee OA	Surgical delivery	Significantly improved pain and joint function	[40]
Infrapatellar fat pad-derived MSCs	1.89 × 10^6^	Platelet-rich plasma (PRP)	16 months	Knee OA	IA injection	Relievedpain in patients with OA	[41]
Allogenic Wharton's jelly umbilical cord MSCs	–	Porcine type I/II collagen matrix scaffold (ChondrO-Gide)	12 months	Knee cartilage injury	Surgical implantation	Induce hyaline-like regeneration	[42]
Allogenic placental MSCs	0.5–0.6 × 10^8^	–	24 weeks	Knee OA	IA injection	Safe and clinical symptom improvement	[43]
Synovial MSCs	4.7 × 10^7^	–	48 months	Knee cartilage defects	Arthroscopic transplantation	Significantly improved MRI, histologic features, and clinical scores	[44]

BMSCs, bone-marrow-derived MSCs; ADSCs, adipose-derived stem cells; hUC-MSCs, human umbilical-cord-derived MSCs; hUCB-MSCs, human umbilical cord blood-derived MSCs; MRI, magnetic resonance imaging; VAS, visual analog scale; IKDC, International Knee Documentation Committee; KOOS, Knee Injury and Osteoarthritis Outcome Score; KSS, Knee Society Score; WOMAC, Western Ontario and McMaster Universities Osteoarthritis Index; OA, osteoarthritis; IA, intra-articular.

### Mesenchymal stromal cell homing

When tissues and organs are injured, natural repair mechanisms are activated to release MSCs into circulation that migrate to the damaged tissue sites and secrete powerful immunomodulatory, angiogenic, and anti-apoptotic factors to create a regeneration-promoting microenvironment [45–48]. For cartilage defects, including OA, the tissue has been reported to regenerate via homing of endogenous cells. Synovial tissue can potentially recruit endogenous stem cells, which facilitate partial tissue regeneration even in the absence of exogenous cell transplantation.

Exogenously transplanted MSCs also tend to migrate into tissues and affect tissue regeneration. The use of exogenously transplanted stem cells as "biological regeneration supplements" is largely based on their natural abilities to mobilize, migrate, and home. Mechanisms of cell migration and “nesting” into injury sites mediated by a broad range of chemokine and growth factor receptors are primarily relevant to MSCs [49, 50]. The most well-studied examples include stromal cell-derived factor 1 (SDF1) and its receptor and CXC-chemokine receptor 4 (CXCR4) implicated in MSC homing [51]. SDF-1 has been shown to be upregulated at injury sites and to affect MSC migration in a dose-dependent manner. Therefore, SDF-1 has been used to induce migration and homing of MSCs to cartilage defect sites for enhancing tissue repair [52, 53]. Other chemokine-chemokine receptor pairs, including PDGF-PDGFR, SCF-c-Kit, HGF-c-Met, VEGF-VEGFR, MCP-1-CCR2 and HMGB1-RAGE, are also involved in MSC recruitment and migration [54]. Thus, MSCs can be modulated for therapeutic purposes,, and external cues can enhance their homing efficiency towards damaged tissues.

**Fig. 1:**
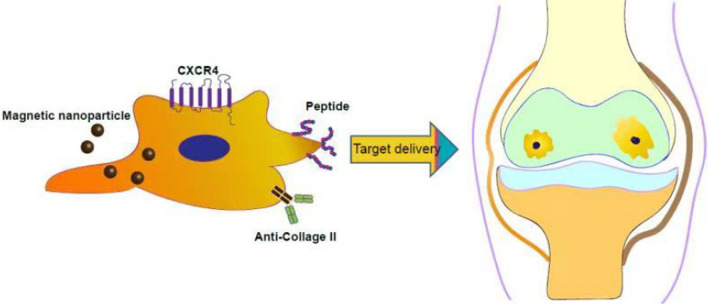
Targeted delivery of modified MSCs for cartilage repair

Pretreatment of MSCs with specific compounds, cytokines, and hypoxic conditions can enhance cell migration toward the injury site. Increased expression of the cytokine membrane receptor (CXCR4) can be induced by stimulation with Fms-like tyrosine kinase (Flt-3) ligands, stem cell factor (SCF), interleukin (IL), or hepatocyte growth factor (HGF) [55]. Preconditioning of MSCs with tumor necrosis factor alpha (TNFα) can improve the migration of MSCs to the site of injury and affect osteoclast function [56]. Similarly, MSC preconditioning with insulin growth factor-1 (IGF-1) has been shown to increase the expression of CXCR4 and improve cell migration capacity in vitro and in vivo [41,[57]. Other small molecules, such as glycogen synthase kinase-3β inhibitors, lithium, and the histone deacetylase inhibitor valproate, can effectively enhance MSC migration ability by upregulating the expression of CXCR4 and matrix metalloproteinases (MMP) [58] (See Fig. 1).

Another strategy to improve MSC homing ability is to use genetic manipulation to increase the expression of targeted molecules. Many research groups have reported that CXCR4 overexpression exhibits variable efficiency in increasing the targeting potential of MSCs.

### Engineered MSCs for targeted therapy

**Fig. 2:**
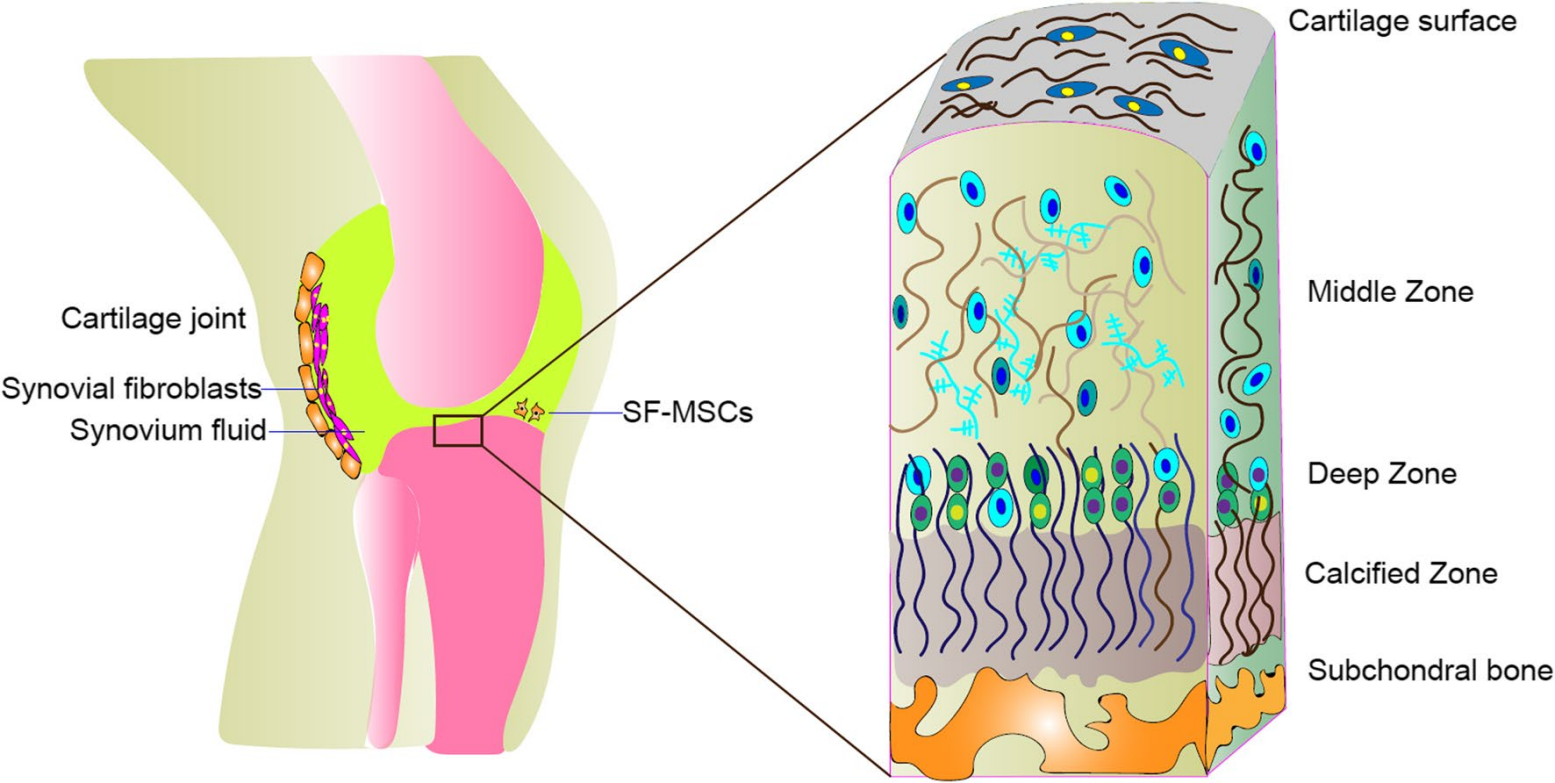
Morphology of the cartilage tissue. The superficial zone consists of a high concentration of collagen fibers parallel to the articular surface. This layer is rich in type II collagen and contains small amounts of type I collagen and proteoglycans. The middle zone is the thickest cartilage layer and accounts for 40–60% of articular cartilage volume. Collagen fibers in this area are thicker and contain higher levels of proteoglycans. The deep region has the highest concentration of proteoglycans where chondrocytes and collagen fibers are arranged in vertical columns perpendicular to the surface.. In areas of calcification, a proteoglycan-free matrix surrounds round chondrocytes with hypertrophic phenotypes

Although endogenous homing mechanisms help MSCs reach and engraft at the target site, most MSCs fail to attach to the damaged cartilage layer. The fate of MSCs following intra-articular delivery is still unclear due to the vigorous metabolism of synovial fluid in the joint cavity. It is possible that after MSCs are injected into the joint cavity, they quickly spread into systemic circulation due to the rapid turnover of synovial capillaries and lymphatic vessels, resulting in only the transient presence of MSCs in the joint cavity. Moreover, the abundance of anionic proteoglycans in the cartilage layer endows the tissue matrix with high density and a high negative fixed charge density, making it extremely challenging to retain MSCs in the cartilage tissue [59]. As illustrated in Fig. 2, the multi-zonal structure of AC makes it quite difficult for MSCs to penetrate through the cartilage surface to reach the tissue zones**.**

**Fig. 3:**
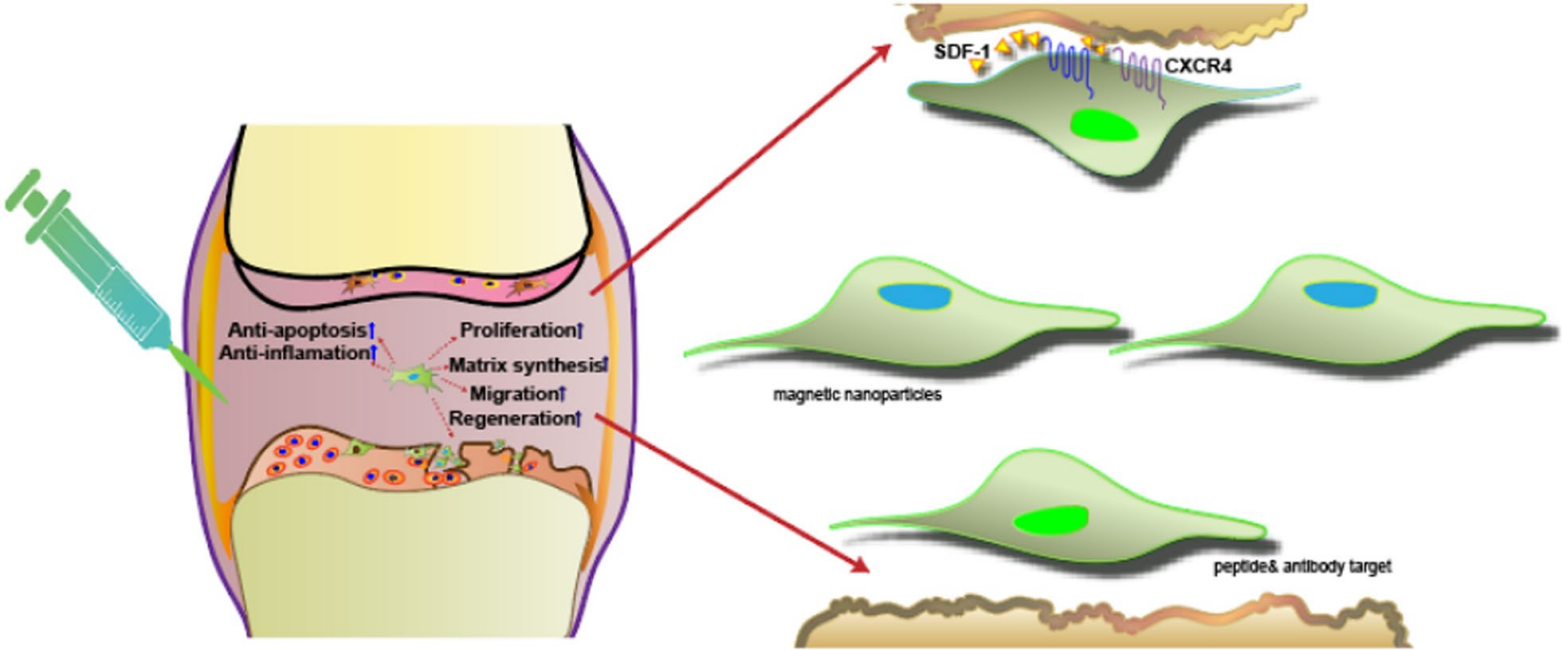
Strategies for the targeted delivery of MSCs to articular cartilage. IA of MSCs showed both tissue regeneration potential and paracrine effects (e.g., anti-apoptosis, reduced inflammation, or regulation of immune responses). MSC surfaces can be conjugated with specific antibodies against antigens present on the cartilage surface (e.g., anti- cartilage matrix proteoglycan, Col II) (Abs). Surface modification of MSCs via cartilage matrix-targeting peptides imparts additional functionality to enable site-specific delivery. Genetically engineered MSCs highly expressing CXCR4 facilitate MSC recruitment to the cartilage via a homing mechanism. In addition, magnetic targeting systems can direct the delivery of MSCs to the desired region by using external magnetic forces

MSCs can be genetically modified virally and non-virally to overexpress therapeutic proteins and targeting moieties. In addition, chemical conjugation, non-covalent interactions, and enzymatic modifications have been used to coat MSC membranes with targeting groups (Fig. 3). MSCs can also be treated with non-peptide drugs or magnetic nanoparticles to enhance their efficacy and targeted delivery.

### Genetically modified MSCs for targeted therapy

Overexpression of chemokine receptors such as CXCR1, CXCR4, or CXCR7 has been shown to enhance the migration and targeting ability of MSCs [60]. Cho, et al. claimed that intravenous infusion of autologous MSCs overexpressing CXCR4 significantly inhibited bone loss in an OVX-induced mouse model. Furthermore, MSCs overexpressing RANK-Fc effectively enhance bone-protective effects [61]. In a mouse model of myocardial infarction, increased CXCR4 expression can induce migration toward the infarction site, improving cardiac performance [62]. These studies indicated the value of enhancing CXCR4 expression to regulate MSC trafficking.

While non-viral methods are preferred, particularly in the context of potential clinical applications, they remain limited due to their low transfection efficiency. Several cationic liposomal reagents (such as IBAfect, a polycationic liposomal transfection reagent) have been used to achieve superior CXCR4 transfection efficiency [63].

### Surface engineering of MSCs by antibodies for targeted therapy

Biomedical engineering offers new opportunities for surface modification of living cells with antibodies. Various antibodies can be applied to cell surfaces as uniform ultrathin coatings via hydrophobic interactions, covalent binding, or lipid-PEG methodology. After functionalization with antibody conjugation, the cell surface can bind to a specific antigen on the target tissue.

In the lipidation method, palmitate-conjugated protein A/G is bound to the Fc region of the Ab [58]. Palmitate-derivatized antibodies against vascular adhesion molecules ICAM-1, VCAM-1 and MAdCAM-1 have been shown to enhance the homing of surface-engineered cells [64–66]. Dennis et al. embedded lipidated protein G into the membranes of chondrogenic progenitor cells, allowing subsequent binding of anchor protein G antibodies to cartilage matrix antigens on the extracellular surface. Cells coated with multiple antibodies were found to preferentially adhere to cartilage repair sites when added to rabbit cartilage explants [67]. Modifying the cell membrane with palmitate-conjugated type II collagen enables efficient targeted delivery of therapeutic MSCs to the osteochondral defect explant [64]. These results suggest that coating cell membranes with antibodies against matrix molecules effectively promotes the adhesion of MSCs to specific cartilage damage locations.

### Peptide functionalization of MSCs for targeted modification

Cell-homing peptides (CHPs) are highly specific affinity peptides that target the cell surface. Several research groups have exploited CHPs for cartilage treatment. Previously, a self-assembling peptide (SAP) functionalized with the bone marrow homing peptide (BMHP) motif SKPPGTSS was designed to regulate MSC homing and promote the repair of cartilage defects after microfracture [68].

Pi et al. identified a short CHP sequence with a high binding affinity toward chondrocytes. They introduced a non-viral vector in which fluorescently labeled chondrocyte-affinity peptide (CAP) was covalently bound to polyethyleneimine (PEI) and injected into rabbit knees to target hyaline cartilage. The results using fluorescein isothiocyanate (FITC)-labeled CAP-PEI entered into the chondrocytes. demonstrating cartilage-specific targeting [69].

In a recent report, we also demonstrated that a CAP-modified exosome could deliver drugs to chondrocytes in joints, alleviating OA in a rat model [70, 71]. Similarly, Cheung et al. used phage display screening technology to identify two cartilage-binding peptide sequences of 12 amino acids in length that specifically bound cartilage ECM and chondrocytes, showing that these polypeptides adhered strongly to the surface of chondrocytes [72]. Recently, Sangar. et al. identified a cystine-dense peptide (CDP) that rapidly accumulated in the cartilage after systemic injection. The accumulating peptide CDP-11R reached the articular cartilage layer within 30 min and wa detectable for more than 4 days [73].

A domain in placental growth factor-2 (PlGF-2(123-144)) was found to bind ECM proteins with high affinity [74]. As the cartilage tissue is rich in ECM proteins, using engineered TNF α conjugated with the PlGF-2123-144 peptide could enhance local retention time in the cartilage [75]. Similarly, a recent study by Delint et al. had rationally designed a nanocomplex composed of PlGF-2 fused to the supercharged green fluorescent protein (scGFP). The complex was then electrostatically coupled to anionic polymer surfactant chains to generate oxidized poly-oxyethylene non-ylphenyl ether (S-) scGFP_PIGF2 nanocomplexes, which were spontaneously inserted into the plasma membrane of hMSCs. Their findings indicated that PIGF nanocomplex-modified hMSCs had significantly increased affinity for collagen II, a cartilage ECM component, and high concentrations of hMSCs were detected at the cartilage interface [76]. Thus, modification of hMSC membranes with scGFP_PlGF2 can improve the efficacy of stem cell-based injection therapies for damaged articular cartilage.

Bifunctional peptide-modified functional ferritin is another example developed to promote BMSC engraftment for cartilage regeneration. Researchers engineered ferritin nanocages containing RGD peptides that could target BMSCs and WYRGRL peptides with intrinsic affinity for the cartilage matrix component of collagen II [77]. The combination of these two significant peptides enabled the recruitment of exogenous MSCs to areas of defective cartilage. In Table 2, we have listed all ligands that can be used for cartilage-targeted MSC delivery.

**Table 2:** Cartilage-specific targeting ligands for stem cell delivery

Ligand	Target	Application	Ref
Cartilage penetrating cationic peptide (CPC)	Fixed charge density (FCD) of cartilage	Rapid penetration in full cartilage, high absorption, and 7-day retention of CPC + 14	[78]
Supercharged green fluorescent proteins (GFPs)	Cartilage	Rapid transport into full-thickness cartilage and chondrocyte	[79]
CDP-11R	Cartilage	Accumulation in the cartilage after systemic intravenous injection; alleviation 0f joint inflammation and off-target toxicity	[73]
CBP peptide: LRELHLNNNC	Collagens	Targeting the extracellular matrix of inflamed tissues	[80]
PIGF2_123–144	ECM, collagen type II	Improved cartilage adhesion of MSCs	[74–76]
CAP peptide: DWRVIIPPRPSA	Chondrocytes	Plasmid DNA and exosome target delivery	[69–71]
Aggrecan-binding peptides peptide: RLDPTSYLRTFW, HDSQLEALIKFM	Aggrecan	Binding to chondrocytes and extracellular matrix	[72]
Type II collagen binding peptide: WYRGRL	Collagen type 2 (CII)	Deep zone retention, increased half-life and retention in the cartilage	[77, 81–83]
P15-1 peptide: STMMSRSHKTRSHHV	Hyaluronan (HA)	Inhibition of chondrocytes inflammation	[84]
Monoclonal antibody (mAbCII)	Collagen type 2	Enhanced collagen II binding and MMP-13 siRNA delivery for OA therapy	[85]
Avimer M26	Collagen II	Enhanced cartilage retention time	[86]
Multi-arm Avidin (mAv)	Aggrecan-associated glycosaminoglycans (GAGs)	Penetration through the full thickness of cartilage	[87, 88]

The advantages of CHP include high targeting specificity, ease of synthesis, small size, low molecular weight, and high biocompatibility. Attaching multiple ligands simultaneously to the cell surface or other carriers is possible. The unique ability of CHP to target specific tissues makes them promising candidates for cellular delivery in clinical settings. Especially for AC, chondrocyte-homing peptides can be integrated onto the surface of MSCs to deliver therapeutic MSCs throughout diseased tissues. Although MSC surface engineering approaches have great therapeutic potential, they may alter MSC membrane properties. Also, the associated biosafety issues limit their clinical applications. For example, covalent anchoring of peptides or Abs to the MSC surface may interfere with membrane protein function and affect signaling pathways, resulting in aberrant ligand-receptor binding and may alter cell fate.

### Magnetic stem cell targeting

For magnetic MSC delivery, magnetically loaded cells are administered to target areas with the assistance of a magnetic field. MSCs typically internalize nanoparticles by passive diffusion or endocytosis upon adding magnetic nanoparticles (MNPs) to the cell culture medium. Some commonly used MNPs, such as nickel and cobalt, may be somewhat toxic to the cells and for in vivo applications. However, iron oxide magnetite (Fe_3_O_4_) and maghemite (γ-Fe_2_O_3_) have been identified as biocompatible MNPs. Compared to magnetite MNPs, maghemite MNPs cause less damage to recipient cells due to the oxidized state of iron (Fe^3+^). In preclinical studies, magnetic stem cell targeting has been used to concentrate MSCs in bone or cartilage tissue [89].

We have developed magnetic nanocomposite-combined MSCs for the treatment of cartilage defects. Stem cell differentiation was promoted by exposure to a pulsed electromagnetic field, which has broad applications in cartilage tissue engineering [90–92]. Kobayashi et al. labeled BMSCs with Feridex and injected them into rabbit and pig models of osteochondral defect, showing enhanced engraftment into the chondral defect under external magnetic force [93]. It was demonstrated that besides improved MSC proliferation due to magnetic labeling with ferucarbotran, targeted delivery of MSCs to the injury site using an external magnetic device resulted in complete repair and integration of the targeted tissue. Thus, MSC delivery using a magnetic targeting system has the potential to overcome barriers inhibiting the repair of severe chronic osteochondral defects. Furthermore, delivery of magnetically labeled MSCs to target tissues allows their retention in the cartilage defect area long enough to repair full-thickness cartilage defects in a mini-pig model [94].

A clinical study evaluated the safety and efficacy of magnetic targeting of MSCs in patients with focal cartilage defects in the knee joint. Autologous bone marrow MSCs were magnetized with ferucarbotran and injected into the knee joint in the presence of a 1.0 Tesla (T) magnetic force. No serious adverse events were observed during magnet-targeted therapy. After 48 weeks of treatment, MRI showed that the cartilage defect area was almost completely filled with cartilage-like tissue [95]. These findings suggest that magnetic targeting of MSCs is safe and significantly improves clinical outcomes and, therefore can be used as a minimally invasive treatment for cartilage repair.

MNPs that are sufficiently small (between 10 and 30 nm) can exhibit superparamagnetic behavior, and such superparamagnetic nanoparticles (SPIONs) are important materials for potential clinical applications of enhanced MSC-based cell therapy. Furthermore, they can be used for MSC labeling and as in vivo tracking agents due to the strong signals they generate under MRI. For example, SPION-ASC-labeled ASCs were successfully tracked by MRI following injection into the knee joint. The implanted ASCs adhered to the injured meniscus and differentiated into meniscus tissue under the action of a permanent external magnet [96].

In recent years, the concept of a magnetic microrobot has been proposed. Under the action of a magnetic field, magnetically driven microrobot-targeted cell delivery could significantly improve the low targeting efficiency of MSCs to promote tissue regeneration [97]. A microrobot loaded with human adipose-derived MSCs was guided by a magnetic field to specific lesions in rabbit knee cartilage to stimulate regeneration. The microrobot degraded within three weeks without causing inflammation in rabbits, indicating good biocompatibility and biodegradability [98]. The applications of magnetically targeted MSCs to animal models and clinical studies are summarized in Table 3.

**Table 3:** Applications of magnetically targeted delivery of MSCs for articular cartilage repair

Model	MSC donor	Nanoparticles	External magnet	Ref
In vivo*:* rabbit and pig models of osteochondral defects	hBMSCs	Ferumoxide (Felidex®)	Magnetic force (0.6 T)	[93]
In vivo*:* pig model of full-thickness cartilage defect	MSCs	Magnetic hydrogels	Magnetic force (1.5 T) for 10 min	[94]
In vivo*:* human articular cartilage defect	hBMSCs	Ferucarbotran (Resovist®)	1.0-T compact magnet for10min	[95]
In vivo*:* rabbit model of a massive meniscal defect	Rabbit ADSCs	Ferucarbotran	Permanent magnet	[96]
In vivo*:* knee cartilage defect model	hADSCs	Microrobot (Feraheme)	N.A	[99]
In vivo*:* rabbit model of osteochondral defect	Rabbit BMSCs	Ferucarbotran	External magnetic device	[100]
In vivo*:* rat model of sub-chronic skeletal muscle injury	hMSCs	Ferucarbotran	Magnetic strength (1.5 T) for 10 min	[101]
In vivo*:* rat model of femoral fracture	Rat BMSCs	Ferucarbotran	Magnetic strength (5.07 T) for 10 min and 60 min	[102]
In vivo*:* rabbit ulnar defect	Rabbit BMSC	Ferumoxide	Magnetic strength (1.5 T) for 10 min	[103]
Ex vivo*:* porcine knee osteochondral defect implanted with hMSCs	hMSCs	Ferumoxide	N. A	[104]
Ex vivo*:* human cartilage	hBMSC	Ferumoxide	Magnetic force (0.4 or 0.6 T) for 6 h	[105]
Ex vivo*:* human osteochondral defects	MSCs	N-dodecyl-poly-ethylenimine-coated SPION ∼50–110 nm	Magnetic force (0.57 T)	[99]

## Conclusions and perspectives

MSCs have been widely used in cartilage repair due to their self-renewing pluripotency and differentiation ability. Over the past few decades, MSC-based therapies have emerged as promising new therapeutics in regenerative medicine. While the results of clinical studies have been very positive, some inconsistent data have emerged from Phase I/II trials. Intra-articular injection of MSCs results in limited cell retention and survival in the cartilage. Therefore, the cartilage regeneration capacity of exogenous MSCs following transplantation is limited. Modification strategies can be combined with compounds that enhance MSC survival, migration, homing, and adhesion to optimize cell survival and maximize therapeutic efficacy. Also, the route of administration, number of modified cells administered, and engraftment frequency require further improvement.

In evaluating the fate and efficacy of MSCs, innovative in vivo imaging strategies and quantitative assays are critical to determining MSC distribution, viability, and function. In addition, using appropriate ex vivo cartilage and animal models can provide further insight into pharmacokinetics and pharmacodynamics under specific pathological conditions. Overall, cell-based targeted therapies represent a major new development direction for accelerating the clinical translation of MSCs to treat cartilage diseases.

## Data Availability

Not applicable.

## Abbreviations

OA: Osteoarthritis
MSC: Mesenchymal stem cells
IA: Intra-articular
AC: Articular cartilage
CPSCs: Cartilage-derived stem/progenitor cells
UC-MSCs: Umbilical cord MSCs
BMSCs: Bone marrow-derived MSCs
ADSCs: Adipose tissue-derived stem cells
SDSCs: Synovial tissue-derived stem cells
PB-MSCs: Peripheral blood-derived MSCs
hADMSCs: Human adipose-derived MSCs
SPIONs: Superparamagnetic nanoparticles
PRP: Platelet-rich plasma
SDF1: Cell-derived factor 1
CXCR4: CXC-chemokine receptor 4
TNFα: Tumor necrosis factor alpha
IGF-1: Insulin growth factor-1
MMP: Matrix metalloproteinases
MNPs: Magnetic nanoparticles
CHPs: Cell homing peptides
GFP: Green fluorescent protein
CDP: cystine-dense peptide
CPC: Cartilage penetrating cationic peptide
CAP: Chondrocyte-affinity peptide
